# Effects of First-Time Overnight CPAP Therapy for Increasing the Complexity of the Patient's Physiological System

**DOI:** 10.1155/2014/652012

**Published:** 2014-07-21

**Authors:** Hsien-Tsai Wu, Hong-Ruei Chen, Wen-Yao Pan, Cyuan-Cin Liu, Mao-Chang Su, Meng-Chih Lin

**Affiliations:** ^1^Department of Electrical Engineering, National Dong Hwa University, Hualien 97401, Taiwan; ^2^Division of Pulmonary and Critical Care Medicine, Department of Internal Medicine, Kaohsiung Chang Gung Memorial Hospital and Chang Gung University College of Medicine, Kaohsiung 83301, Taiwan; ^3^Sleep Center, Kaohsiung Chang Gung Memorial Hospital and Chang Gung University College of Medicine, Kaohsiung 83301, Taiwan

## Abstract

Studies regarding the effects of short-term continuous positive airway pressure (CPAP) therapy are not sufficient. A total of 35 patients with moderate to severe untreated OSA were divided into 2 groups. Group 1 comprised 22 patients who underwent polysomnography (PSG) for one night, and Group 2 comprised 13 patients who received PSG combined with CPAP therapy. To evaluate the influence of receiving CPAP therapy for one night, we measured 5 min wrist pulse signals before and after the experiment to assess heart rate variability, as well as novel short time multiscale entropy (sMSE) indicator that examines complexity in physiological signals. The results show that the participants in Group 1 exhibited significant changes in normalized low-frequency power/normalized high-frequency power (nLF/nHF) (0.72 ± 0.09 versus 1.11 ± 0.11, *P* = 0.006) values before and after the PSG study. By contrast, the participants in Group 2 showed no significant changes in the 3 indicators. Regarding the sMSE indicator, Group 2 patients exhibited significant increases in the sMSE. CPAP therapy administered for one night can reduce the sympathovagal imbalance in patients with moderate to severe untreated OSA and increase the complexity of the patient's physiological system, thereby reflecting their overall improved health.

## 1. Introduction

Obstructive sleep apnea (OSA) is widely considered a risk factor for cardiovascular disease [1]. Patients with OSA have a substantially higher likelihood of experiencing a stroke, coronary artery disease, acute myocardial infarction, congestive heart failure, and cardiac arrhythmia [2–7]. In addition, OSA can cause autonomic function abnormalities, which are associated with the risks of cardiovascular disease and increased mortality [8]. Continuous positive airway pressure (CPAP) therapy is currently the main noninvasive OSA treatment method. According to a previous long-term study that monitored patients for 7 years, the probability of developing cardiovascular disease was significantly higher for incompletely treated OSA patients compared to that for efficiently treated OSA patients [9]. In addition, long-term CPAP therapy has been verified to effectively improve OSA patients' vascular functions and reduce their autonomic function abnormalities [10–16].

To emphasize the treatment effects of overnight CPAP therapy for severe OSA patients, Kufoy et al. [17] conducted an experiment for 2 consecutive nights on subjects who had not received CPAP therapy. On the first night, only polysomnography (PSG) measurements were conducted; on the second night, both PSG and CPAP therapy were administered to the patients. Kufoy et al. then analyzed the time-domain HRV of the R wave to R wave interval (RRI) series measured by the electrocardiogram (ECG) obtained from both nights separately. The results indicated that with overnight CPAP therapy, the patients' standard deviation of normal-to-normal intervals (SDNN) in the all-night RRI series increased significantly [17]. This was the first indication of the significant therapeutic effect that only a single course of CPAP therapy has for patients with untreated OSA. However, a more convenient indicator is required to evaluate the level of overall health improvement for patients who receive only a single course of CPAP therapy.

Recently, numerous translational medicine studies have indicated that information implied by the changing complexity of physiological signals can be used to evaluate the overall health of a human body [18–21]. As stated in the related literature, the human body's physiological signals are influenced by multiple temporal and spatial scales and exhibit fluctuating complexity characteristics. The degree of complexity fluctuation declines with age, disease, and aggravation of disease [19, 21]. Thus, we proposed the following 2 basic hypotheses: (1) OSA patients administered only PSG without treatment will experience declining complexity fluctuations in physiological signals because of repeated apnea and hypoxia during all-night sleep, and (2) patients administered PSG and CPAP therapy simultaneously will experience increased complexity fluctuations in physiological signals because of improved apnea or hypopnea.

Therefore, this study administers first-time and one-night CPAP therapy to patients with moderate to severe untreated OSA and explores the improvement of autonomic functions and overall health status after one-night CPAP treatment.

## 2. Materials and Methods

### 2.1. Study Population

This study was reviewed and approved by the Institutional Review Board of Chang Gung Memorial Hospital, Kaohsiung. Data collection and measurements were conducted at the hospital's Sleep Center from December 2010 to February 2012. The participant exclusion criteria were healthy subjects or patients with mild OSA who indicated an apnea-hypopnea index (AHI) of less than 15 events per hour, patients who had previously undergone CPAP therapy or otolaryngologic surgery, and patients with a history of cardiac arrhythmias, congestive heart failure, myocardial infarctions, ischemic heart disease, stroke, diabetes mellitus, and other sleep disorders, such as central sleep apnea (CSA), restless limb syndrome (RLS), periodic limb movement disorder (PLMD), and chronic obstructive pulmonary disease (COPD). After screening subjects using these criteria, we recruited 35 patients with moderate to severe untreated OSA. The subjects were divided into 2 groups, where 22 received only baseline PSG (Group 1) and the other 13 with similar OSA severity revealed by previous baseline PSG receiving first-time CPAP titration therapy (Group 2).

### 2.2. PSG and CPAP Treatment

A Sandman SD32+ Digital Amplifier (Embla, Colorado, USA) was used to conduct PSG measurements. The participants' sleep stage was recorded at 30-s intervals, and respiratory events were identified by experienced technicians [22]. Apnea was defined as the absence of airflow for at least 10 s. Hypopnea was defined as a 50% or more reduction of the respiratory airflow and a 3% or more decline in blood oxygen concentrations, or the wakening from sleep caused by these conditions. AHI was defined as the total number of apneas and hypopneas per hour of sleep. We performed CPAP therapy using an automatic pressure adjusting machine GoodKnight 420E (Nellcor Puritan Bennett, California, USA). The total sleep time for all subjects was at least 4 h. Therapy was considered effective for subjects who simultaneously received PSG measurements and CPAP therapy if they showed an AHI of less than 5 events per hour.

### 2.3. Study Protocol

The period for PSG data collection and measurement was from 10:00 PM to 06:00 AM the next morning. Before PSG, we conducted questionnaire surveys with the subjects regarding their basic information and disease history and requested that they complete an informed consent form. Subsequently, the physicians explained the experimental procedure to the subjects. Finally, experienced sleep technicians prepared and installed instruments for the PSG measurements and CPAP therapy. The subjects of Group 1 received standard PSG measurements, whereas the subjects in Group 2 received both PSG measurements and CPAP therapy. After the examination instruments were set up, the patient lied down in a supine position quietly and awake for at least 5 min. Then 5 min of stable pulse signals were obtained for analysis using an air pressure sensing system (APSS) as previously described [23, 24]. The pulse signals in the next morning were obtained in the same manner just after the sleep study was conducted.

### 2.4. Heart Rate Variability and Short Time Multiscale Entropy Computation

We applied a Fast Fourier transform (FFT) to the extracted 5-min pulse signals and computed the frequency-domain HRV parameters based on different frequency bands. Total power was defined as the energy between 0 and 0.4 Hz. The very low-frequency power (<0.04 Hz, VLF), low-frequency power (0.04–0.15 Hz, LF), high-frequency power (0.15–0.4 Hz, HF), and normalized LF power were calculated based on the LF/(total power − VLF), and the normalized HF was calculated using the HF/(total power − VLF) [25]. Extant studies have indicated that nLF is related to sympathetic activity, nHF is related to vagal activity, and LF/HF represents sympathovagal balance [26]. Therefore, this study used the nLF, nHF, and LF/HF indicators to evaluate changes in the autonomic functions of the subjects before and after CPAP therapy.

The short time multiscale entropy (sMSE) is a modified MSE [18, 21] approach of computation that enables the use of large scale factor for analysis on data acquired through a shortened time period. The basic concept is the creation of different time series through removing a small number of recordings from the beginning without affecting the overall trend and complexity of the acquired signals. The acquired time series then undergo sample entropy (*S*_*E*_) [18] computation with steady values of entropy obtained.

Through altering the number of lag from 0 to *L* (where *L* = *τ* − 1, *τ* = coarse-grained scale factor) on the native time series in (1), a new time series, *T* ^(*p*)^, can be obtained in (2). Thus, the number of new time series generated is *L* + 1:

(1) $$\begin{array}{c} T_{N}=\left\{X_{1},X_{2},\ldots ,X_{N-1},X_{N}\right\}, \end{array}$$

(2) T(p)={Xk,Xk+1,Xk+2,…,XN−1,XN},k=p+1, p=0,1,2,…,L.

The *L* + 1 time series acquired then undergo coarse-grained processing with a scale factor *τ* in (3), giving the time series of *y* ^(*p*)(*τ*)^. Hence,

(3) yj(p)(τ)=1τ∑k=(j−1)τ+1+pjτ+pXk,1≤j≤⌊N−Pτ⌋, p=0,1,2,…,L.

The *L* + 1*y* ^(*p*)(*τ*)^ are then subjected to sample entropy computation and averaged, giving MSE_*τ*_ of scale factor *τ* in

(4) $$\begin{array}{c} {\text{sMSE}}_{\tau }=\frac{1}{L+1}\overset{L}{\underset{p=0}{\sum }}{\text{S}}_{E}\left(y^{\left(p)(\tau \right)}\right). \end{array}$$

### 2.5. Statistical Analysis

The results are expressed as mean ± standard error (SE). All statistical tests were performed using SPSS software, version 14.0 (SPSS Inc., Chicago, IL). The differences between Groups 1 and 2 were assessed using a Mann-Whitney *U* test. The differences in the HRV and sMSE parameters before and after the experiment were examined using a Wilcoxon test. Statistical significance was defined as *P* < 0.05.

## 3. Results

Table 1 shows the demographic information of the subjects in Groups 1 and 2. No statistical differences in age, height, weight, BMI, neck circumference, and waist circumference were observed between the 2 groups. Regarding the baseline AHI, although the subjects in Group 2 possessed a higher AHI compared to that of the subjects in Group 1 (62.68 ± 5.77 versus 51.60 ± 5.75), no statistical difference was exhibited (*P* = 0.212).

**Figure 1:**
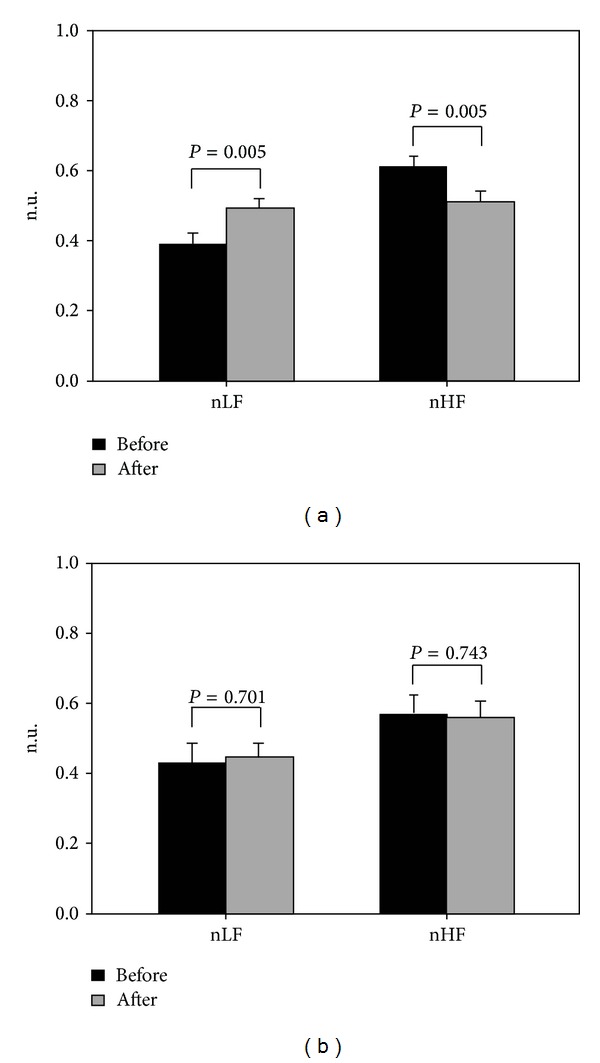
Patients with moderate to severe OSA were divided into 2 groups, and their autonomic activity changes before and after the sleep experiment were compared using the nLF and nHF indicators. (a) Group 1 received PSG measurements only and (b) Group 2 received PSG measurements and CPAP therapy simultaneously.

Figure 1 shows the changes in nLF and nHF for Groups 1 and 2 before and after sleep.  Figure 1(a) shows that Group 1 experienced a significant pre- to postexperiment increase in the nLF indicator (0.39 ± 0.03 versus 0.49 ± 0.03, *P* = 0.005), whereas nHF declined significantly (0.61 ± 0.03 versus 0.51 ± 0.03, *P* = 0.005).  Figure 1(b) shows that Group 2 did not exhibit significant pre- and postexperiment changes in the nLF (0.43 ± 0.05 versus 0.44 ± 0.04, *P* = 0.701) and nHF (0.57 ± 0.05 versus 0.56 ± 0.04, *P* = 0.743) indicators.

**Figure 2:**
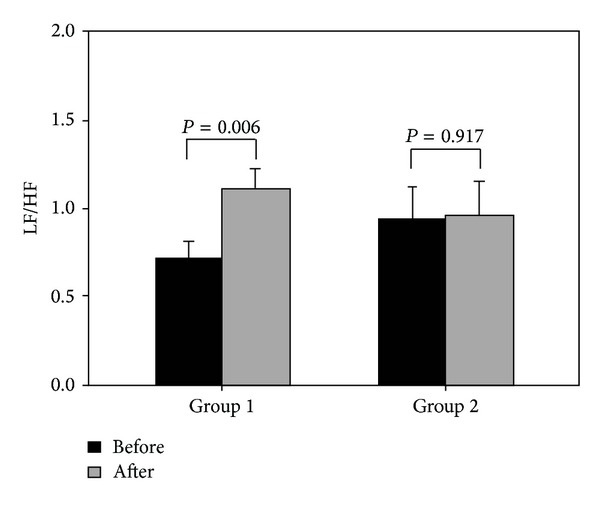
The sympathovagal balance changes of the subjects in Groups 1 and 2 before and after the sleep experiment.

Figure 2 shows the changes in LF/HF for Groups 1 and 2 before and after the sleep experiment. Group 1 exhibited a significant increase in LF/HF after the experiment (0.72 ± 0.09 versus 1.11 ± 0.11, *P* = 0.006), whereas Group 2 showed no significant difference in their pre- and postexperiment measurements (0.94 ± 0.18 versus 0.96 ± 0.19, *P* = 0.917).

Table 2 shows the comparison of sMSE values in each scale for Groups 1 and 2 before and after the experiment. For Group 1, only sMSE_1_ declined significantly (1.89 ± 0.07 versus 1.69 ± 0.07, *P* = 0.017); the other scales exhibited no significant changes. Regarding the pre- and postexperiment differences for Group 2, the sMSE_1_ (1.61 ± 0.08 versus 1.76 ± 0.07, *P* = 0.002), sMSE_2_ (1.70 ± 0.06 versus 1.89 ± 0.08, *P* = 0.013), and sMSE_3_ (1.65 ± 0.06 versus 1.73 ± 0.06, *P* = 0.039) indicators showed significant increases.

## 4. Discussion

In Western countries, approximately 4% of middle-aged men and 2% of middle-aged women develop OSA [27]. In recent years, people in Taiwan have developed an understanding of OSA and have begun emphasizing this condition. Based on the situations we encountered at the hospital's outpatient visits and the Sleep Center, most patients were unaware of their symptoms. These patients only sought treatment because their partner or others had discovered the occurrence of apnea or severe snoring when they slept, or because they experienced symptoms such as day-time hypersomnia. Because they could not perceive these symptoms, the majority of the patients did not realize that they had moderate or even severe OSA until they underwent PSG examinations. However, determining the presence and severity of OSA requires a full night of PSG examinations. Additionally, patients are often required to wait for 3 to 6 months to receive PSG examinations due to the fact that there are only a limited number of instruments and resources in Taiwan. Nevertheless, when a OSA diagnosis is confirmed, the majority of patients are administered PSG and CPAP therapy simultaneously to observe the therapeutic effects of CPAP therapy and thus allows physicians to advise patients regarding subsequent treatments. Therefore, we collected and analyzed data from patients with untreated OSA. The patients were divided into Group 1 (first-time PSG examination) and Group 2 (second-time PSG examination with CPAP therapy). The frequency-domain HRV parameters and the sMSE indicator were used to analyze whether autonomic and complexity changes were exhibited by the 2 groups of subjects after undergoing CPAP therapy.

Because the subjects in Group 1 received PSG only, they continued to experience repeated apnea or hypopnea during sleep, which led to intermittent hypoxia and sleep fragmentation. Consequently, these patients were susceptible to symptoms such as higher blood pressure [28, 29], increased oxidative stress [30–32], and excessive sympathetic activity [33, 34].  Figure 1(a) shows that the subjects in Group 1 exhibited a significant increase in nLF (representing sympathetic activity) after one night. By contrast, the nHF (representing vagal activity) decreased significantly. These phenomena not only correspond to that reported by previous studies [33], but they also indicate that the subjects in Group 1 experienced sympathovagal imbalance caused by substantial changes in sympathetic and vagal activities after one night of repeated apnea or hypopnea (Figure 2).

Previous studies have confirmed that long-term CPAP therapy can reduce the risk of developing cardiovascular disease [9, 35]. Drager et al. [10] reported that the intima-media thickness (IMT) and carotid-femoral pulse wave velocity (cfPWV) indicators of patients with severe OSA who received 4 months of CPAP therapy exhibited a significant decline compared to that for patients who did not receive CPAP therapy. In addition, CPAP therapy can effectively reduce OSA patients' blood pressure [36, 37] and improve their sympathovagal balance [13, 15, 34]. These studies not only verified that long-term CPAP therapy improves the physical health of patients with OSA but also explored the influence that long-term CPAP therapy has on other aspects, such as vascular health and nervous system function. Unlike most studies investigating the long-term effects of CPAP therapy, Kufoy et al. found that overnight CPAP therapy can significantly alter the SDNN indicator of patients with severe OSA. Therefore, based on this finding, we used frequency-domain HRV and the sMSE indicator to analyze the subjects in Group 2. For measurement convenience, to conserve time and to prevent different sleep stages from influencing the HRV analysis [38], we did not analyze the subjects' overnight RRI series as Kufoy et al. did; instead, we extracted 5 min of stable pulse signals before and after sleep. The signals were used to evaluate whether CPAP therapy caused between-subject differences.

Our results indicated that the subjects in Group 2 showed no significant changes in sympathetic and vagal activities following a single night of CPAP therapy (Figure 1(b)). The sympathovagal indicator of LF/HF also remained unchanged (Figure 2). This result showed that CPAP therapy effectively inhibited the excessive sympathetic activity induced by hypoxia compared to that for the subjects in Group 1 [34]. Therefore, we found that first-time and overnight CPAP therapy can improve patients' sympathovagal imbalance, which is an effect similar to that of long-term therapy [13, 15, 34]. Furthermore, a previous study conducted an experiment where CPAP therapy was discontinued for 2 weeks for patients with OSA undergoing long-term therapy [39]. The results showed that the patients' morning heart rate had increased significantly on day 1 and continued rising over time without the therapy. The patients' sympathetic activity also exhibited a gradual increase. Combining our findings with those reported by extant study [39], we contended that the first night of CPAP therapy can improve patients' sympathovagal imbalance. This reinforces the importance of regularly receiving CPAP therapy, which helps physicians advise OSA patients to receive CPAP therapy and increases patient willingness to undergo regular long-term CPAP therapy.

In addition to employing traditional frequency-domain HRV parameters to explore changes in autonomic functions, we introduced the concept of complexity in physiological signals. A number of studies have indicated that the information implied by these signals can be used to evaluate the overall health of our physiological systems [18–21]. The sMSE algorithm, which integrates multiscale and sample entropy concepts, used in this study was previously employed for research regarding various diseases, such as Alzheimer's disease [40, 41], heart failure [19, 42], and diabetes mellitus [21, 43]. These studies have confirmed that the effects of disease on physical health can result in decreasing complexity. Furthermore, an extant study applied sample entropy to RRI series during sleep to evaluate the HRV changes of OSA patients and healthy people. The results not only indicated that OSA patients' sample entropy values were lower than those of healthy people [44], but also showed that the deterioration in the OSA patients' overall health status according to the previous studies [18, 19]. Meanwhile, sMSE was used to evaluate the complexity fluctuations in the cardiovascular systems of patients who underwent carotid stenting surgery [45]. Through sMSE, the scholars observed a significant increase in the patients' 1-h postoperative and 1-day postoperative physiological complexities. However, conventional HRV parameters cannot show postoperative changes. This explains why when evaluating changes in the cardiovascular system, sMSE provides more information compared to conventional HRV parameters, and can accurately reflect rapid and drastic changes in the cardiovascular system. The results in Table 2 show that the subjects in Group 1 exhibited a significant decline in sMSE_1_ after the experiment. By contrast, the subjects in Group 2 who received CPAP therapy exhibited significant increases in MSE_1_, sMSE_2_, and sMSE_3_. Such phenomena support the hypotheses proposed in this study and imply that the administration of CPAP therapy can induce variations in the sMSE_2_ and sMSE_3_ indicators. This also reflects the possible correlation of sMSE_2_ and sMSE_3_ with the interaction between heart rate and respiration [18, 19]. From the perspective of complexity, the decreasing complexity of the subjects in Group 1 can be considered a deterioration in their overall health status. Conversely, the subjects in Group 2 exhibited substantially increased complexity after receiving CPAP therapy. This can be explained as follows: because CPAP therapy reduces sleep apnea, patients' overall health status is improved.

## 5. Study Limitations

This study had a number of limitations that are worth highlighting: (1) because of the Sleep Center's limited number of available instruments and related resources and strict exclusion criteria, only 35 patients were included in this study. (2) By attaching PSG measurement pads and wires to the subjects, we extracted and analyzed 5 min of stable pulse signals before and after the experiment to reduce the experiment time and avoid placing a greater burden on the subjects after one night of experiment. Although 5 min of data is relatively limited compared to the data of other studies, the results can provide an equal reflection of the differences between the patients who received CPAP therapy and the patients who did not. (3) According to our knowledge, this study was the first to evaluate the degree to which CPAP therapy improves OSA patients' overall health status using the sMSE algorithm. Although the experimental results validated the 2 hypotheses proposed in this study and evidently showed the differing trends corresponding to the presence and absence of CPAP therapy, complexity comparisons with patients presenting mild OSA and healthy people before and after receiving or not receiving CPAP therapy were not conducted in this study. Thus, we hope to recruit patients with varying severities of OSA and healthy people to enlarge the sample size in future studies. We are keen to further explore the physiological indicators related to complexity and apply these indicators to clinically evaluate the therapeutic effects of CPAP therapy for patients.

## 6. Conclusions

Based on the results of this study, we derived the following 2 primary findings: (1) considering autonomic functions, patients who received PSG without CPAP therapy experienced significantly increased sympathetic activity, leading to an autonomic function imbalance, identical to the results of previous studies. By contrast, CPAP therapy for only one night, which is a comparatively short time, was found to have reduced the patients' sympathovagal imbalance abnormalities. (2) Regarding the sMSE indicator for evaluating the overall health status, overnight CPAP therapy improved patients' health status and increased the complexity of their physiological signals. Conversely, patients who did not receive CPAP therapy exhibited a decline in complexity.

## Figures and Tables

**Table 1:** Basic participant information.

	Group 1	Group 2	*P* value
Number	22	13	N/A
Age (years)	50.32 ± 2.44	53.77 ± 3.95	0.437
Height (m)	1.68 ± 0.01	1.68 ± 0.02	0.839
Weight (kg)	76.73 ± 2.64	85.39 ± 3.84	0.064
BMI (kg/m^2^)	27.20 ± 0.85	30.02 ± 1.17	0.056
Neck circumference (cm)	40.01 ± 0.68	41.57 ± 0.76	0.074
Waist circumference (cm)	97.65 ± 2.33	104.69 ± 3.03	0.153
AHI (events/hour)	51.60 ± 5.75	62.68 ± 5.77	0.212

Data are expressed as mean ± SE. Variables were compared by using Mann-Whitney *U* test, with a *P* < 0.05 showing statistical significance. BMI: body mass index, AHI: apnea-hypopnea index.

**Table 2:** Differences in the sMSE indicator before and after the sleep experiment.

	Group 1			Group 2		
	Before sleep	After sleep	*P* value	Before sleep	After sleep	*P* value
sMSE_1_	1.89 ± 0.07	1.69 ± 0.07	0.017	1.61 ± 0.08	1.76 ± 0.07	0.002
sMSE_2_	1.88 ± 0.07	1.76 ± 0.07	0.158	1.70 ± 0.06	1.89 ± 0.08	0.013
sMSE_3_	1.71 ± 0.07	1.63 ± 0.06	0.115	1.65 ± 0.06	1.73 ± 0.06	0.039

Data are expressed as mean ± SE. Variables were compared by using Wilcoxon test, with a *P* < 0.05 showing statistical significance.

sMSE_1_: Sample entropy for each of the coarse-grained time series can be obtained and plotted against the scale factor 1.

sMSE_2_: Sample entropy for each of the coarse-grained time series can be obtained and plotted against the scale factor 2.

sMSE_3_: Sample entropy for each of the coarse-grained time series can be obtained and plotted against the scale factor 3.

## Abbreviations

CPAP: Continuous positive airway pressure
OSA: Obstructive sleep apnea
PSG: Polysomnography
AHI: Apnea hypopnea index
HRV: Heart rate variability
sMSE: Short time multiscale entropy
nLF: Normalized low-frequency power
nHF: Normalized high-frequency power.
